# ﻿Morphology and multigene phylogeny reveal four new *Xylaria* (Xylariales, Xylariaceae) species from karst region in China

**DOI:** 10.3897/mycokeys.108.130565

**Published:** 2024-09-04

**Authors:** Wenyu Zeng, Kamran Habib, Xin Zhou, Yulin Ren, Xiangchun Shen, Bei Wang, Yingqian Kang, Jichuan Kang, Qirui Li

**Affiliations:** 1 State Key Laboratory of Functions and Applications of Medicinal Plants, Guizhou Medical University, Gui’an New District, 561113, China; 2 The High Efficacy Application of Natural Medicinal Resources Engineering Centre of Guizhou Province (The Key Laboratory of Optimal Utilization of Natural Medicine Resources), School of Pharmaceutical Sciences, Guizhou Medical University, Gui’an New District, 561113, China; 3 Department of Botany, Khushal Khan Khattak University, Karak, KP, Pakistan; 4 Shandong Qidu Pharmaceutical Co., Ltd, Zibo City, 255400, China; 5 Key Laboratory of Environmental Pollution Monitoring and Disease Control, Ministry of Education, School of Basic Medical Sciences, Guizhou Medical University, Gui’an New District, 561113, China; 6 Engineering and Research Centre for Southwest Bio‐Pharmaceutical, Resources of National Education Ministry of China, Guizhou University, Guiyang, 561113, China

**Keywords:** Folicolous fungi, Fungal systematics, Southwest China, Xylariaceae, taxonomy

## Abstract

This study presents the identification of four novel *Xylaria* species, discovered in the karst region of China. The discovery was facilitated by a rigorous analysis that encompassed both morpho-anatomical features and multi-locus phylogenetics utilizing sequences from the ITS, *rpb*2, and *TUB*2 loci. The newly identified species are designated as *Xylariajichuanii***sp. nov.**, *X.nanningensis***sp. nov.**, *X.orientalis***sp. nov.**, and *X.taiyangheensis***sp. nov.** The distinction of these species from their known counterparts was verified through comparison of morphological features and phylogenetic analysis. The study further provides detailed morphological descriptions, illustrative representations, and a phylogenetic tree, all of which contribute to the taxonomic positioning of these novel species.

## ﻿Introduction

Karst is a geological formation characterized by surface and subsurface features that result from the dissolution of soluble rocks by water. This includes morphological, hydrological, and hydrogeological elements (Marčić et al. 2022). Karst landscapes exhibit distinctive shapes, arising from a unique combination of morphological, hydrological, and hydrogeological features of the surface and subsurface, all rooted in water-soluble rocks. These karst regions and their caves constitute invaluable natural resources, harbouring a broad spectrum of ecological niches, many of which are often singular in nature (Pipan and Culver 2013; Forti 2015). Karst regions boast remarkable biodiversity, characterized by endemic species that are uniquely endemic to their respective locales. The diverse habitats, unique microclimates, and isolation of karst regions contribute to this exceptional biological diversity (Jakob et al. 2022; Marčić et al. 2022; Melekhina et al. 2022; Dong et al. 2023; Mičetić 2023; Zhang et al. 2023). Within karst regions, speciation and the existence of discontinuous populations of organisms with phylogenetically distinct origins are commonplace. Being inaccessible, karst landscapes often serve as natural refuges for species that have vanished elsewhere due to hunting and habitat destruction (Li et al. 2021; Marasinghe et al. 2022; Hyde et al. 2023). These ecosystems, despite being biodiversity hotspots, remain largely unmapped. Consequently, identifying biodiversity and understanding the ecology of karst habitats is paramount, given their sensitivity to disturbance and the challenges associated with restoration efforts (Mičetić 2023).

The genus *Xylaria*, a member of the Xylariaceae family, comprises a highly diverse group of fungi that possess significant ecological significance. Extensive research has underscored the genus’s key role as wood decomposers (Rogers 1986; Fournier et al. 2020; Ma et al. 2022a), a valuable source of bioactive secondary metabolites (Healy et al. 2004), and as endophytes residing within diverse plant species (Davis et al. 2003; Promputtha et al. 2007). Notably, *Xylaria* has been proven to synthesize a range of bioactive compounds exhibiting promising potential as antibiotic agents (Elias et al. 2018), thereby establishing its pharmaceutical relevance. These fungi are commonly encountered in temperate, subtropical, and tropical regions across the globe, frequently associated with wood, fallen fruits or seeds, leaves or petioles, and termite nests (Dennis 1956; Rogers and Samuels 1986; San and Rogers 1989; Ju and Hsieh 2007; Fournier 2014). Species within the genus are distinguished by their upright, stipitate, and charred stromata, characterized by perithecia that are entirely immersed (San and Rogers 1989; Stadler et al. 2013; Konta et al. 2020; Samarakoon et al. 2022). Globally, there have been reports of more than 300 species belonging to the genus *Xylaria* (Kirk et al. 2008), while Index Fungorum lists an even more extensive catalog of more than 800 epithets (Ma et al. 2022a). Only in China, including the karst areas of south-western China, about 115 species of woody plants have been recorded (Ma et al. 2022a; Li et al. 2024a), indicating that this genus has rich diversity and universality.

In this study, we collected *Xylaria* specimens from fallen leaves and twigs of plants in the Karst regions of two neighboring provinces Yunnan and Guangxi Zhuang Autonomous Region, China. We performed comprehensive morphological examinations and phylogenetic analyses to ascertain species identification and their positions within the phylogenetic tree. Our phylogenetic analysis utilized sequences from the ITS, *rpb*2, and *TUB*2 loci, employing both maximum likelihood and Bayesian frameworks. The results distinguished these specimens from other known species within the genus, leading us to propose them as new species.

## ﻿Materials and methods

### ﻿Sample collection

The specimens were collected during surveys conducted in the Karst regions including Yunnan province and Guangxi Zhuang Autonomous Region of China during 2022. All related collection information, including collection time, collector, altitude, latitude and longitude, etc, were recorded. The photos of the collected materials were taken using a Canon G15 camera (Canon Corporation, Tokyo, Japan). Materials were placed in paper bags and taken to the lab for examination. They were dried using a portable fan drier. The specimens were ready for both morphological and molecular studies. The dried specimens were carefully labelled and stored in an ultra-low freezer at -80 °C for one week to eliminate any insects and their eggs. The cultures were obtained before the -80 °C treatment. All specimens were deposited at the herbarium of Guizhou Medical University (GMB) and the Herbarium of Cryptogams, Herbarium of Kunming Institute of Botany, Chinese Academy of Sciences (KUN-HKAS). Living cultures were deposited at the Guizhou Medical University Culture Collection (GMBC).

### ﻿Morphological characterization and isolation

Morphological characteristics of specimens were examined, and photomicrographs were taken as described in Wu et al. (2022), Senanayake and Calabon (2020). Macroscopic characteristics were observed under an Olympus SZ61 stereomicroscope and photographed with a Canon 700D digital camera fitted to a light microscope (Nikon Ni). Samples for microscopic examination were mounted in distilled water and Melzer’s reagent. More than 30 ascospores and 30 asci were measured for each sample using the Tarosoft image framework (v. 0.9.0.7). The images were arranged using Adobe Photoshop CS6 (Adobe Systems, USA). Cultures were obtained from single spores method described in Senanayake and Calabon (2020). Germinating spores were observed with a Stereo Zoom microscope and transferred to potato dextrose agar (PDA; 39 g/l distilled water, Difco potato dextrose). The cultures were incubated at 25–30 °C for 4–6 weeks, with frequent observations.

### ﻿DNA extraction, Polymerase Chain Reaction (PCR) amplification and sequencing

Mycelium was scraped from pure culture plates using a sterilized scalpel and was used for DNA extraction with the methods of the BIOMIGA fungus genomic DNA extraction kit. For some specimens where the ascospores did not germinate, we used a method of directly extracting DNA from the contents of the perithecium. The DNA samples were kept at –20 °C. Internal transcribed spacers (ITS), TUB2 (β-tubulin), and RNA polymerase II second largest subunit (rpb2) were amplified by PCR with primers ITS1/ITS4 (White et al. 1990; Gardes and Bruns 1993), Bt2a/Bt2b or T1/T22 (Glass and Donaldson 1995; O’Donnell and Cigelnik 1997), and RPB2-5F/RPB2-7cR (Liu et al. 1999) respectively. The components of a 25 μL volume PCR mixture were: 9.5 μL of double distilled water, 12.5 μL of PCR Master Mix, 1 μL of each primer, and 1 μL of template DNA. Qualified PCR products were checked through 1.5% agarose gel electrophoresis stained with GoldenView, and were sent to Sangon Co., China, for sequencing (Xie et al. 2020).

### ﻿Sequence alignments and phylogenetic analyses

All the obtained sequences were deposited in the GenBank (Table 1). The molecular phylogeny was inferred from a combined dataset of ITS, *TUB*2 and *rpb*2 sequences. The reference sequences retrieved from open databases originated from latest *Xylaria* articles (Hsieh et al. 2010; Stadler et al. 2013; Li et al. 2024a; etc) and the Blastn results of close matches and additional Xylariaceae representatives. Sequences were aligned using the MAFFT v.7.110 online program (Katoh et al. 2019) with the default settings, respectively. The alignment was adjusted manually using BioEdit v.7.0.5.3 (Hall 1999) were necessary, and used trimAl to select available DNA sequences. The maximum likelihood (ML) analysis was implemented in RAxML v.8.2.12 using the GTRGAMMA substitution model with 1,000 bootstrap replicates (Stamatakis 2014). Phylogenetic analyses were conducted using Bayesian inference in MrBayes v. 3.2.1 (Ronquist et al. 2012) online, with Markov chain Monte Carlo (MCMC) sampling in MrBayes v.3.2.2 (Ronquist et al. 2012) used to calculate posterior probabilities (PP). Six simultaneous Markov chains were run for 1,000,000 generations, and trees were sampled every 1,000^th^ generation. The convergence of the MCMC procedure was assessed from the effective sample size scores (all > 100) using MrBayes. The first 25% of the trees were discarded as burn-ins. The remainder was used to calculate the posterior probabilities (PPs) for individual branches. The phylogenetic tree was visualized in FIGTREE v.1.4.3 (Rambaut 2012). All analyses were run on the CIPRES Science Gateway v 3.3 webportal (Miller et al. 2010). The alignments are available in TreeBASE (www.treebase.org/treebase-web/home.html) under ID 31594 for ITS, *TUB*2 and *rpb*2 sequences.

**Table 1.** List of taxa used for the phylogenetic tree. GenBank accession numbers, specimen numbers, country and reference are given. Holotype specimens are labelled with HT. Ex-type cultures are labelled with ET. Species highlighted in bold were derived from this study. N/A: not available.

Species	Specimen No.	Country	Reference	GenBank accession numbers		
				ITS	*rpb*2	*TUB*2
*Albicollumberberidicola*	WU:MYC 0043994(HT)	Greece	Voglmayr et al. (2022)	ON869278	ON808457	ON808501
*Albicollumcanicolle*	WU:MYC 0043997(ET)	Spain	Voglmayr et al. (2022)	ON869279	ON808458	ON808502
*Albicollumlongisporum*	WU:MYC 0044004(HT)	Spain	Voglmayr et al. (2022)	NR182514	ON808465	ON808509
*Albicollumvincensii*	WU:MYC 0044014(ET)	Austria	Voglmayr et al. (2022)	ON869297	ON808475	ON808519
*Albicollumvincensii*	WU:MYC 0044017	Spain	Voglmayr et al. (2022)	ON869298	ON808476	ON808520
*Amphiroselliniafushanensis*	HAST 91111209(HT)	China	Hsieh et al. (2010)	GU339496	GQ848339	GQ495950
*Amphirosellinianigrospora*	HAST 91092308(HT)	China	Hsieh et al. (2010)	GU322457	GQ848340	GQ495951
*Astrocystisbambusae*	HAST 89021904	China	Hsieh et al. (2010)	GU322449	GQ844836	GQ495942
*Astrocystisconcavispora*	MFLUCC:14-0174	Italy	Daranagama et al. (2015)	KP297404	KP340532	KP406615
*Astrocystismirabilis*	HAST 94070803	China	Hsieh et al. (2010)	GU322448	GQ844835	GQ495941
*Brunneiperidiumgracilentum*	MFLUCC:14-0011	Italy	Daranagama et al. (2015)	KP297400	KP340528	KP406611
*Collodisculabambusae*	GZ-62	China	Li et al. (2015)	KP054279	KP276675	KP276674
*Collodisculajaponica*	CBS 124266	China	Jaklitsch and Voglmayr (2012)	JF440974	KY624273	KY624316
*Daldinialoculatoides*	CBS 113279(HT)	UK	Vu et al. (2019)	MH862918	KY624247	KX271246
*Dematophorabuxi*	JDR 99	France	Hsieh et al. (2010)	GU300070	GQ844780	GQ470228
*Dematophoranecatrix*	CBS 349.36	Argentina	Peláez et al. (2008)	AY909001	KY624275	KY624310
*Entoleucamammata*	JDR 100	France	Hsieh et al. (2010)	GU300072	GQ844782	GQ470230
*Entalbostromaerumpens*	ICMP21152	New Zealand	Johnston et al. (2016)	NR154013	KX258204	KX258205
*Haloroselliniakrabiensis*	MFLU 17-2596(HT)	Thailand	Dayarathne et al. (2020)	MN047119	N/A	MN431493
*Haloroselliniarhizophorae*	MFLU 17-2591	Thailand	Dayarathne et al. (2020)	MN047118	N/A	MN431492
*Haloroselliniaxylocarpi*	MFLU 18-0545(HT)	Thailand	Dayarathne et al. (2020)	MN047120	N/A	MN077076
*Helicogermslitaclypeata*	MFLU 18-0852(HT)	Thailand	Samarakoon et al. (2022)	MW240666	MW658647	MW775614
*Hypocoprazeae*	MFLU 18-0809(HT)	Thailand	Samarakoon et al. (2022)	MW240671	MW658650	MW775616
*Hypoxylonfragiforme*	MUCL 51264(ET)	Germany	Stadler et al. (2013)	KC477229	KM186296	KX271282
*Jackrogersellacohaerens*	YMJ 310	France	Hsieh et al. (2010)	EF026140	GQ844766	AY951655
*Kretzschmariaclavus*	YMJ 114	French Guiana	Hsieh et al. (2010)	EF026126	GQ844789	EF025611
*Kretzschmariadeusta*	CBS 163.93	Germany	Stadler et al. (2013)	KC477237	KY624227	KX271251
*Kretzschmariafrustulosa*	HAST 92092010	China	Hsieh et al. (2010)	GU322451	GQ844838	GQ495944
*Kretzschmariafrustulosa*	HAST 771	Guadeloupe	Hsieh et al. (2010)	GU322450	GQ844837	GQ495943
*Kretzschmariaguyanensis*	HAST 89062903	China	Hsieh et al. (2010)	GU300079	GQ844792	GQ478214
*Kretzschmarialucidula*	JDR 112	French Guiana	Hsieh et al. (2010)	EF026125	GQ844790	EF025610
*Kretzschmariamegalospora*	JDR 229	Malaysia	Hsieh et al. (2010)	EF026124	GQ844791	EF025609
*Kretzschmarianeocaledonica*	HAST 94031003	China	Hsieh et al. (2010)	GU300078	GQ844788	GQ478213
*Kretzschmariapavimentosa*	JDR 109	China	Hsieh et al. (2010)	GU300077	GQ844787	GQ478212
*Kretzschmariasandvicensis*	JDR 113	USA	Hsieh et al. (2010)	GU300076	GQ844786	GQ478211
*Kretzschmariellaculmorum*	JDR 88	France	Pi et al. (2021)	KX430043	N/A	KX430046
*Leptomassariasimplex*	WU:MYC:0044025	Austria	Voglmayr et al. (2022)	ON869305	ON808483	ON808527
*Leptomassariasimplex*	WU:MYC:0044026	Austria	Voglmayr et al. (2022)	ON869306	ON808484	ON808528
*Linosporopsisischnotheca*	LIF1	Switzerland	Voglmayr and Beenken (2020)	MN818952	MN820708	MN820715
*Linosporopsisischnotheca*	LIF2	Switzerland	Voglmayr and Beenken (2020)	MN818953	MN820709	MN820716
*Linosporopsisochracea*	LIO3	Germany	Voglmayr and Beenken (2020)	MN818958	MN820714	MN820721
*Linosporopsisochracea*	LIO2	Germany	Voglmayr and Beenken (2020)	MN818957	MN820713	MN820720
*Nemaniaabortiva*	BISH 467(HT)	USA	Hsieh et al. (2010)	GU292816	GQ844768	GQ470219
*Nemaniabeaumontii*	HAST 405	Martinique	Hsieh et al. (2010)	GU292819	GQ844772	GQ470222
*Nemaniabeaumontii*	HAST 90080610	China	Hsieh et al. (2010)	GU292818	GQ844771	GQ470221
*Nemaniadiffusa*	HAST 91020401	China	Hsieh et al. (2010)	GU292817	GQ844769	GQ470220
*Nemaniaethancrensonii*	WU:MYC: 0040047(HT)	USA	Voglmayr et al. (2022)	ON869311	ON808489	ON808533
*Nemaniaillita*	JDR 236	USA	Hsieh et al. (2010)	EF026122	GQ844770	EF025608
*Nemaniamacrocarpa*	WSP 265	USA	Hsieh et al. (2010)	GU292823	GQ844776	GQ470226
*Nemaniamaritima*	HAST 89120401(HT)	China	Hsieh et al. (2010)	GU292822	GQ844775	GQ470225
*Nemaniaprimolutea*	HAST 91102001(HT)	China	Hsieh et al. (2010)	EF026121	GQ844767	EF025607
*Nemaniaserpens*	HAST 235	Canada	Hsieh et al. (2010)	GU292820	GQ844773	GQ470223
*Nemaniasphaeriostoma*	JDR 261	USA	Hsieh et al. (2010)	GU292821	GQ844774	GQ470224
*Nemaniauda*	WU:MYC: 0040046	Austria	Voglmayr et al. (2022)	ON869312	ON808488	ON808532
*Neoxylariaarengae*	MFLUCC 15-0292(HT)	Thailand	Konta et al. (2020)	MT496747	MT502418	N/A
*Neoxylariajuruensis*	HAST 92042501	China	Hsieh et al. (2010)	GU322439	GQ844825	GQ495932
*Oligostomainsidiosum*	WU:MYC: 0044034	Austria	Voglmayr et al. (2022)	ON869313	ON808490	ON808534
*Oligostomainsidiosum*	WU:MYC: 0044033(ET)	Austria	Voglmayr et al. (2022)	ON869315	ON808492	ON808536
*Podosordariamexicana*	WSP 176	Mexico	Hsieh et al. (2010)	GU324762	GQ853039	GQ844840
*Podosordariamuli*	WSP 167(HT)	Mexico	Hsieh et al. (2010)	GU324761	GQ853038	GQ844839
*Poroniapileiformis*	WSP 88113001(ET)	China	Hsieh et al. (2010)	GU324760	GQ853037	GQ502720
*Roselliniaaquila*	MUCL:51703	France	Wendt et al. (2018)	KY610392	KY624285	KX271253
*Roselliniacorticium*	MUCL:51693	France	Wendt et al. (2018)	KY610393	KY624229	KX271254
*Rosellinialamprostoma*	HAST 89112602	China	Hsieh et al. (2010)	EF026118	GQ844778	EF025604
*Roselliniamerrillii*	HAST 89112601	China	Hsieh et al. (2010)	GU300071	GQ844781	GQ470229
*Roselliniasanctae-cruciana*	HAST 90072903	China	Hsieh et al. (2010)	GU292824	GQ844777	GQ470227
*Stilbohypoxylonelaeicola*	HAST 94082615	China	Hsieh et al. (2010)	GU322440	GQ844827	GQ495933
*Stilbohypoxylonelaeidis*	MFLUCC 15-0295a(HT)	Thailand	Konta et al. (2020)	MT496745	MT502416	MT502420
*Stilbohypoxylonquisquiliarum*	JDR 172	French Guiana	Hsieh et al. (2010)	EF026119	GQ853020	EF025605
*Stilbohypoxylonquisquiliarum*	HAST 89091608	China	Hsieh et al. (2010)	EF026120	GQ853021	EF025606
*Stromatoneurosporaphoenix*	BCC 82040	Thailand	Becker et al. (2020)	N/A	MT742606	MT700438
*Virgariaboninensis*	JCM 18624	Japan	Nonaka et al. (2013)	AB740956	N/A	N/A
*Virgarianigra*	NBRC 32656	Japan	Nonaka et al. (2013)	AB670717	N/A	N/A
*Waweliaregia*	CBS:110.10	Netherlands	Vu et al. (2019)	MH854595	N/A	N/A
*Xylariaacuminatilongissima*	HAST 623(HT)	China	Hsieh et al. (2010)	EU178738	GQ853028	GQ502711
*Xylariaaethiopica*	YMJ 1136	Ethiopia	Ma et al. (2022a)	MH790445	MH785222	MH785221
*Xylariaalboareolata*	HAST 543	Guadeloupe	Hsieh et al. (2010)	GU300080	GQ844793	GQ478215
*Xylariaallantoidea*	HAST 94042903	China	Hsieh et al. (2010)	GU324743	GQ848356	GQ502692
*Xylariaamphithele*	HAST 529	Guadeloupe	Hsieh et al. (2010)	GU300083	GQ844796	GQ478218
*Xylariaapoda*	HAST 90080804	China	Hsieh et al. (2010)	GU322437	GQ844823	GQ495930
*Xylariaarbuscula*	HAST 89041211	China	Hsieh et al. (2010)	GU300090	GQ844805	GQ478226
Xylariaarbusculavar.plenofissura	HAST 93082814	China	Hsieh et al. (2010)	GU339495	GQ844804	GQ478225
*Xylariaatrodivaricata*	HAST 95052001(HT)	China	Hsieh et al. (2010)	EU178739	GQ853030	GQ502713
*Xylariaatrosphaerica*	HAST 91111214	China	Hsieh et al. (2010)	GU322459	GQ848342	GQ495953
*Xylariabadia*	HAST 95070101	China	Hsieh et al. (2010)	GU322446	GQ844833	GQ495939
*Xylariabambusicola*	WSP 205(HT)	China	Hsieh et al. (2010)	EF026123	GQ844802	AY951762
*Xylariabambusicola*	JDR 162	Thailand	Hsieh et al. (2010)	GU300088	GQ844801	GQ478223
*Xylariabawanglingensis*	GMB1023(HT)	China	Li et al. (2024a)	OR468975	OR753861	OR477223
*Xylariabawanglingensis*	GMB1162	China	Li et al. (2024a)	OR468976	OR753862	OR477224
*Xylariabotryodalis*	GMB1057(HT)	China	Li et al. (2024a)	OR468978	OR753871	OR477225
*Xylariabotryodalis*	GMB1164	China	Li et al. (2024a)	OR468977	OR753872	OR477226
*Xylariabrunneovinosa*	HAST 720(HT)	China	Hsieh et al. (2010)	EU179862	GQ853023	GQ502706
*Xylariacantareirensis*	HAST 526	Guadeloupe	Hsieh et al. (2010)	GU300085	GQ844798	GQ478220
*Xylariacastorea*	PDD 600	New Zealand	Hsieh et al. (2010)	GU324751	GQ853018	GQ502703
*Xylariacf.castorea*	HAST 91092303	China	Hsieh et al. (2010)	GU324752	GQ853019	GQ502704
*Xylariacf.glebulosa*	HAST 431	Martinique	Hsieh et al. (2010)	GU322462	GQ848345	GQ495956
*Xylariacf.heliscus*	HAST 88113010	China	Hsieh et al. (2010)	GU324742	GQ848355	GQ502691
*Xylariacirrata*	HAST 664	China	Hsieh et al. (2010)	KY243920	GQ853024	GQ502707
*Xylariacoccophora*	HAST 786	French Guiana	Hsieh et al. (2010)	GU300093	GQ844809	GQ487701
*Xylariacompunctum*	CBS 359.61	South Africa	Senanayake et al. (2015)	KT281903	KY624230	KX271255
*Xylariacranioides*	HAST 226	China	Hsieh et al. (2010)	GU300075	GQ844785	GQ478210
*Xylariacrinalis*	FCATAS MHX 751	China	Ma and Li (2018)	MF774330	N/A	N/A
*Xylariacrozonensis*	HAST 398	France	Hsieh et al. (2010)	GU324748	GQ848361	GQ502697
*Xylariacubensis*	JDR 860	USA	Hsieh et al. (2010)	GU991523	GQ848365	GQ502700
*Xylariaculleniae*	JDR 189	Thailand	Hsieh et al. (2010)	GU322442	GQ844829	GQ495935
*Xylariacurta*	HAST 494	Martinique	Hsieh et al. (2010)	GU322444	GQ844831	GQ495937
*Xylariacurta*	HAST 92092022	China	Hsieh et al. (2010)	GU322443	GQ844830	GQ495936
*Xylariadadugangensis*	GMB1036(HT)	China	Li et al. (2024a)	OR468979	OR753863	OR504178
*Xylariadiaoluoshanensis*	HAFFR115	China	Pan et al. (2024)	OR702611	N/A	OR726655
*Xylariadiaoluoshanensis*	HAFFR117	China	Pan et al. (2024)	OR702612	OR757125	OR726656
*Xylariadigitata*	HAST 919	Ukraine	Hsieh et al. (2010)	GU322456	GQ848338	GQ495949
*Xylariadiscolor*	YMJ 1280(ET)	USA	Ju et al. (2012)	JQ087405	JQ087411	JQ087414
*Xylariadoupengshanensis*	GMB1037(HT)	China	Li et al. (2024a)	OR468980	OR753864	OR487773
*Xylariadoupengshanensis*	GMB0773	China	Li et al. (2024a)	OR468981	OR753865	OR487774
*Xylariaellisii*	DAOM:628556(HT)	Canada	Ibrahim et al. (2020)	MN218820	MN216186	N/A
*Xylariaenterogena*	HAST 785	French Guiana	Hsieh et al. (2010)	GU324736	GQ848349	GQ502685
*Xylariaescharoidea*	HAST 658(ET)	China	Hsieh et al. (2010)	EU179864	GQ853026	GQ502709
*Xylariafabacearum*	MFLUCC 16-0456(HT)	Thailand	Ma et al. (2022)	NR171104	MT212202	MT212220
*Xylariafabaceicola*	MFLUCC 16-0461(HT)	Thailand	Ma et al. (2022)	NR171103	MT212201	MT212219
*Xylariafeejeensis*	HAST 565	Martinique	Hsieh et al. (2010)	GU322452	GQ848334	GQ495945
*Xylariafeejeensis*	JDR 180	Thailand	Hsieh et al. (2010)	GU322453	GQ848335	GQ495946
*Xylariafeejeensis*	HAST 92092013	China	Hsieh et al. (2010)	GU322454	GQ848336	GQ495947
*Xylariaficicola*	HMJAU 22818	China	Pan et al. (2022)	MZ351258	N/A	N/A
*Xylariafiliformis*	GUM IRN 1052	Iran	Hashemi et al. (2015)	KP218907	N/A	N/A
*Xylariafiliformis*	FCATAS MHX 750	China	Ma and Li (2018)	MF774332	N/A	N/A
*Xylariafimbriata*	HAST 491	Martinique	Hsieh et al. (2010)	GU324753	GQ853022	GQ502705
*Xylariafissilis*	HAST 367	Martinique	Hsieh et al. (2010)	GU300073	GQ844783	GQ470231
*Xylariafulvotomentosa*	HAFFR124	China	Pan et al. (2024)	OR702619	OR757121	OR726658
*Xylariafulvotomentosa*	HAFFR129	China	Pan et al. (2024)	OR702620	OR757122	OR726659
*Xylariaglaucae*	GMB1051(HT)	China	Li et al. (2024a)	OR468984	OR753869	OR484926
*Xylariaglaucae*	GMB1163	China	Li et al. (2024a)	OR468983	OR753870	OR484927
*Xylariaglobosa*	HAST 775	Guadeloupe	Hsieh et al. (2010)	GU324735	GQ848348	GQ502684
*Xylariagrammica*	HAST 479	China	Hsieh et al. (2010)	GU300097	GQ844813	GQ487704
*Xylariagriseosepiacea*	HAST 641(HT)	China	Hsieh et al. (2010)	EU179865	GQ853031	GQ502714
*Xylariaguizhouensis*	GMB1059(HT)	China	Li et al. (2024a)	OR468982	OR753873	OR484928
*Xylariaguizhouensis*	GMB1058	China	Li et al. (2024a)	OR468986	OR753874	OR484929
*Xylariahedyosmicola*	FCATAS856(HT)	China	Pan et al. (2022)	MZ227121	MZ683407	MZ221183
*Xylariahedyosmicola*	FCATAS857	China	Pan et al. (2022)	MZ227023	MZ851780	MZ221184
*Xylariahypoxylon*	HAST 570	Guadeloupe	Hsieh et al. (2010)	GU300101	GQ844817	GQ487708
*Xylariahypoxylon*	JDR 865	Thailand	Hsieh et al. (2010)	GU322432	GQ844818	GQ487709
*Xylariahypoxylon*	HAST 152	Belgium	Hsieh et al. (2010)	GU300096	GQ844812	GQ260187
*Xylariahypoxylon*	HAST 95082001	China	Hsieh et al. (2010)	GU300095	GQ844811	GQ487703
*Xylariaintracolorata*	HAST 90080402	China	Hsieh et al. (2010)	GU324741	GQ848354	GQ502690
*Xylariaintraflava*	HAST 725(HT)	China	Hsieh et al. (2010)	EU179866	GQ853035	GQ502718
*Xylariajaponica*	GMB1079(HT)	China	Li et al. (2024a)	OR468985	OR887270	OR485581
*Xylariajaponica*	GMB1080	China	Li et al. (2024a)	OR468987	N/A	OR485582
***Xylariajichuanii***	**GMB4703(HT)**	**China**	**This study**	**PQ108599**	**N/A**	**PQ106645**
***Xylariajichuanii***	**GMB4707**	**China**	**This study**	**PQ108600**	**N/A**	**PQ106646**
*Xylariajinshanensis*	GMB1067(HT)	China	Li et al. (2024a)	OR468988	OR753876	OR484931
*Xylariajinshanensis*	GMB1165	China	Li et al. (2024a)	OR468989	OR753877	OR484932
*Xylariakarsticola*	MFLU:23-0049	Thailand	Fournier et al. (2011)	OQ457210	OQ597842	OQ601533
*Xylariakaryophthora*	DRH059(HT)	Guyana	Husbands et al. (2018)	KY564220	KY564216	N/A
*Xylariakuankuoshuiensis*	GMB1068(HT)	China	Li et al. (2024a)	OR468990	N/A	OR484933
*Xylarialaevis*	HAST 419	Martinique	Hsieh et al. (2010)	GU324746	GQ848359	GQ502695
*Xylarialaevis*	HAST 95072910	China	Hsieh et al. (2010)	GU324747	GQ848360	GQ502696
*Xylarialindericola*	FCATAS852(HT)	China	Pan et al. (2022)	MZ005635	MZ031982	MZ031978
*Xylarialindericola*	FCATAS853	China	Pan et al. (2022)	MZ005636	MZ048749	MZ031979
*Xylarialiquidambaris*	HAST 93090701	China	Hsieh et al. (2010)	GU300094	GQ844810	GQ487702
Xylarialuteostromatavar.macrospora	HAST 508	Martinique	Hsieh et al. (2010)	GU324739	GQ848352	GQ502688
*Xylariamali*	CBS 385.35	USA	U’Ren et al. (2016)	KU683769	KU684286	KU684205
*Xylariameliacearum*	JDR 148	Puerto Rico	Hsieh et al. (2010)	GU300084	GQ844797	GQ478219
*Xylariamicroceras*	HAST 414	Guadeloup	Hsieh et al. (2010)	GU300086	GQ844799	GQ478221
*Xylariamontagnei*	HAST 495	Martinique	Hsieh et al. (2010)	GU322455	GQ848337	GQ495948
*Xylariamultiplex*	JDR 259	USA	Hsieh et al. (2010)	GU300099	GQ844815	GQ487706
*Xylariamultiplex*	HAST 580	Martinique	Hsieh et al. (2010)	GU300098	GQ844814	GQ487705
*Xylariamuscula*	HAST 520	Guadeloupe	Hsieh et al. (2010)	GU300087	GQ844800	GQ478222
***Xylariananningensis***	**GMB4702(HT)**	**China**	**This study**	**PQ108601**	**PQ106653**	**PQ106647**
***Xylariananningensis***	**GMB4706**	**China**	**This study**	**PQ108602**	**PQ106654**	**PQ106648**
*Xylarianecrophora*	DMCC2127	USA	Garcia-Aroca et al. (2021)	MN846321	MN917805	MN917782
*Xylarianecrophora*	DMCC2477	USA	Garcia-Aroca et al. (2021)	MH046898	MH113626	MH113628
*Xylarianegundinis*	GMB1082(HT)	China	Li et al. (2024a)	OR468993	OR887273	OR485583
*Xylarianegundinis*	GMB1166	China	Li et al. (2024a)	OR468992	OR887274	OR485584
*Xylariaochraceostroma*	HAST 401(HT)	China	Hsieh et al. (2010)	EU179869	GQ853034	GQ502717
*Xylariaoligotoma*	HAST 784	French Guiana	Hsieh et al. (2010)	GU300092	GQ844808	GQ487700
*Xylariaophiopoda*	HAST 93082805	China	Hsieh et al. (2010)	GU322461	GQ848344	GQ495955
*Xylariaorbiculati*	GMB1083(HT)	China	Li et al. (2024a)	OR468995	OR887275	OR485585
*Xylariaorbiculati*	GMB1084	China	Li et al. (2024a)	OR468994	OR887276	OR485586
***Xylariaorientalis***	**GMB4701(HT)**	**China**	**This study**	**PQ108603**	**PQ106655**	**PQ106649**
***Xylariaorientalis***	**GMB4705**	**China**	**This study**	**PQ108604**	**PQ106656**	**PQ106650**
*Xylariaovate*	GMB1085(HT)	China	Li et al. (2024a)	OR468998	OR887277	N/A
*Xylariaovate*	GMB1086	China	Li et al. (2024a)	OR468996	OR887278	N/A
*Xylariaoxyacanthae*	JDR 859	USA	Hsieh et al. (2010)	GU322434	GQ844820	GQ495927
*Xylariapalmicola*	PDD 604	New Zealand	Hsieh et al. (2010)	GU322436	GQ844822	GQ495929
*Xylariapapulis*	HAST 89021903	China	Hsieh et al. (2010)	GU300100	GQ844816	GQ487707
*Xylariapetchii*	HAFFR118	China	Pan et al. (2024)	OR702617	OR757123	OR735172
*Xylariapetchii*	HAFFR126	China	Pan et al. (2024)	OR702618	OR757124	OR735173
*Xylariaphyllocharis*	HAST 528	Guadeloupe	Hsieh et al. (2010)	GU322445	GQ844832	GQ495938
*Xylariapolymorpha*	JDR 1012	USA	Hsieh et al. (2010)	GU322460	GQ848343	GQ495954
*Xylariapolysporicola*	FCATAS848(HT)	China	Pan et al. (2022)	MZ005592	MZ031980	MZ031976
*Xylariapolysporicola*	FCATAS849	China	Pan et al. (2022)	MZ005591	MZ031981	MZ031977
*Xylariapseudoanisopleura*	GMB1088(HT)	China	Li et al. (2024a)	N/A	OR887279	OR485587
*Xylariapseudobambusicola*	GMB1090(HT)	China	Li et al. (2024a)	OR469002	OR887280	OR485590
*Xylariapseudobambusicola*	GMB1091	China	Li et al. (2024a)	OR469004	OR887281	OR485591
*Xylariapseudocubensis*	GMB1089(HT)	China	Li et al. (2024a)	OR468997	OR887282	OR485588
*Xylariapseudocubensis*	GMB0775	China	Li et al. (2024a)	OR468999	OR887283	OR485589
*Xylariapseudoglobosa*	GMB1092(HT)	China	Li et al. (2024a)	OR469001	OR887284	OR485592
*Xylariapseudohemisphaerica*	GMB1093(HT)	China	Li et al. (2024a)	N/A	OR887285	OR485593
*Xylariapseudohypoxylon*	GMB1094(HT)	China	Li et al. (2024a)	OR469003	OR887286	OR485594
*Xylariapseudohypoxylon*	GMB0776	China	Li et al. (2024a)	OR469005	OR887287	OR485595
*Xylariapuerensis*	GMB1095(HT)	China	Li et al. (2024a)	OR469008	OR887288	OR485596
*Xylariapuerensis*	GMB1167	China	Li et al. (2024a)	OR469007	OR887289	OR485597
*Xylariaqianensis*	GMB1050(HT)	China	Li et al. (2024a)	OR469006	OR753867	OR484924
*Xylariaqianensis*	GMB1049	China	Li et al. (2024a)	OR469013	OR753868	OR484925
*Xylariaqiongzhouensis*	GMB1096(HT)	China	Li et al. (2024a)	OR469009	OR887290	OR485598
*Xylariaqiongzhouensis*	GMB1097	China	Li et al. (2024a)	N/A	OR887291	OR485599
*Xylariareevesiae*	HAST 90071609	China	Hsieh et al. (2010)	GU322435	GQ844821	GQ495928
*Xylariaregalis*	HAST 920	India	Hsieh et al. (2010)	GU324745	GQ848358	GQ502694
*Xylariarogersii*	FCATAS913	China	Ma et al. (2022a)	MZ648825	MZ707119	MZ695799
*Xylariarogersii*	FCATAS915(HT)	China	Ma et al. (2022a)	MZ648827	MZ707121	MZ695800
*Xylariaschimicola*	FCATAS896(HT)	China	Ma et al. (2022a)	MZ648850	MZ707114	MZ695787
*Xylariaschweinitzii*	HAST 92092023	China	Hsieh et al. (2010)	GU322463	GQ848346	GQ495957
*Xylariascruposa*	HAST 497	Martinique	Hsieh et al. (2010)	GU322458	GQ848341	GQ495952
*Xylariashuqunii*	GMB1105(HT)	China	Li et al. (2024a)	OR469012	OR887299	OR485603
*Xylariashuqunii*	GMB1106	China	Li et al. (2024a)	OR469011	OR887300	OR485604
*Xylariasicula*	HAST 90071613	China	Hsieh et al. (2010)	GU300081	GQ844794	GQ478216
*Xylariasinensis*	GMB1109(HT)	China	Li et al. (2024a)	OR469010	OR887301	OR485607
*Xylariasinensis*	GMB0778	China	Li et al. (2024a)	OR469014	OR887302	OR485608
*Xylariastriata*	HAST 304	China	Hsieh et al. (2010)	GU300089	GQ844803	GQ478224
***Xylariataiyangheensis***	**GMB4704(HT)**	**China**	**This study**	**PQ108605**	**PQ106657**	**PQ106651**
***Xylariataiyangheensis***	**GMB4708**	**China**	**This study**	**PQ108606**	**PQ106658**	**PQ106652**
*Xylariatelfairii*	HAST 90081901	China	Hsieh et al. (2010)	GU324738	GQ848351	GQ502687
*Xylariatheaceicola*	FCATAS903(HT)	China	Ma et al. (2022a)	MZ648848	MZ707115	MZ695788
*Xylariatongrenensis*	GMB1169	China	Li et al. (2024a)	OR469016	OR887304	OR485610
*Xylariatuberoides*	HAST 475	Martinique	Hsieh et al. (2010)	GU300074	GQ844784	GQ478209
*Xylariaumbellata*	GMB1116(HT)	China	Li et al. (2024a)	OR469019	OR887305	OR485611
*Xylariaumbellata*	GMB1170	China	Li et al. (2024a)	OR469020	OR887306	OR485612
*Xylariavenosula*	HAST 94080508	USA	Hsieh et al. (2010)	EF026149	GQ844806	EF025617
*Xylariavenustula*	HAST 88113002	China	Hsieh et al. (2010)	GU300091	GQ844807	GQ487699
*Xylariavivantii*	HAST 519	Martinique	Hsieh et al. (2010)	GU322438	GQ844824	GQ495931
*Xylariawallichii*	FCATAS923	China	Ma et al. (2022a)	MZ648861	MZ707118	MZ695793
*Xylariaxishuiensis*	GMB1120(HT)	China	Li et al. (2024a)	OR469021	N/A	OR485613
*Xylariaxishuiensis*	GMB0779	China	Li et al. (2024a)	OR469023	N/A	OR485614
*Xylariayumingii*	GMB1128(HT)	China	Li et al. (2024a)	OR469022	N/A	OR485618
*Xylariayunnanensis*	GMB1129(HT)	China	Li et al. (2024a)	OR469026	OR887310	OR485619
*Xylariayunnanensis*	GMB0780	China	Li et al. (2024a)	OR469025	OR887311	OR485620
*Xylariazangmui*	GMB1130(HT)	China	Li et al. (2024a)	OR469024	OR753880	OR485621
*Xylariazangmui*	GMB0781	China	Li et al. (2024a)	OR469028	OR753881	OR485622
*Xylariazonghuangii*	GMB1131(HT)	China	Li et al. (2024a)	OR469030	OR753878	OR485623
*Xylariazonghuangii*	GMB1132	China	Li et al. (2024a)	OR469027	OR753879	OR485624
*Xylosphaeraberteroi*	JDR 256	USA	Hsieh et al. (2010)	GU324750	GQ848363	GQ502698
*Xylosphaeraberteroi*	HAST 90112623	China	Hsieh et al. (2010)	GU324749	GQ848362	AY951763
*Xylosphaeraberteroi*	MFLUCC 14-0150	Thailand	Ma et al. (2022b)	MZ463147	MZ970707	MZ998966
*Xylosphaeraianthinovelutina*	HAST 553	Martinique	Hsieh et al. (2010)	GU322441	GQ844828	GQ495934

## ﻿Results

### ﻿Phylogenetic analysis

**Figure 1.**
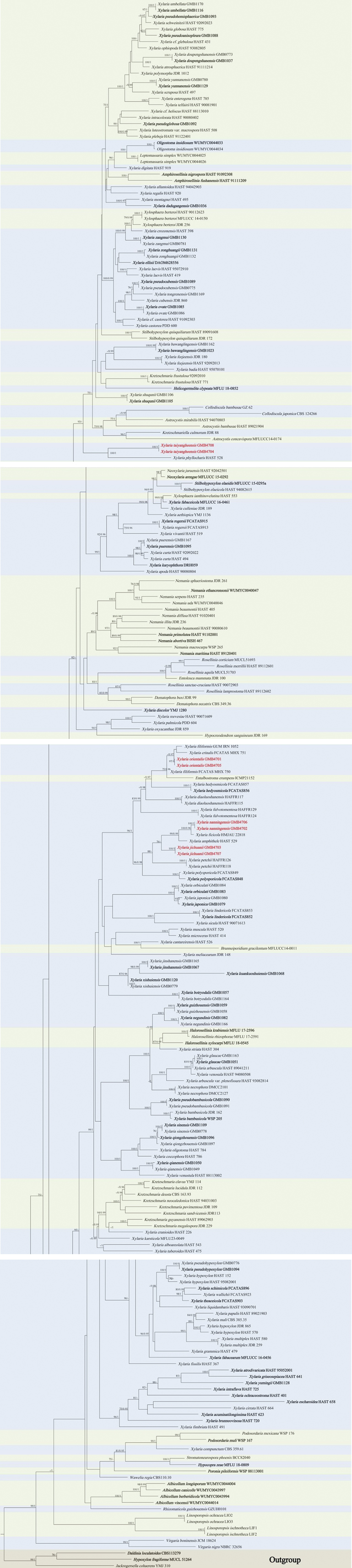
RAxML tree based on a combined ITS, *TUB*2 and *rpb*2 gene sequences data set. Bootstrap support values for maximum likelihood (ML) >75% and Bayesian posterior probabilities (BPP) > 0.95 are displayed above or below the respective branches (ML/BI). The newly described species are marked and red. Type materials were marked bold.

After exclusion of ambiguously aligned regions and long gaps, the final combined data matrix contained 2,320 characters. *Jackrogersellacohaerens* (Pers.) L. Wendt, Kuhnert & M. Stadler, *Hypoxylonfragiforme* (Pers.) J. Kickx f. and *Daldinialoculatoides* Wollw. & M. Stadler were added as the outgroups (Li et al. 2024a; Pan et al. 2022, 2024). The tree topology derived from Maximum Likelihood (ML) analysis closely resembled that of Bayesian Inference (BI) analysis. The best scoring RAxML tree is shown in Fig. 1.

In the phylogram (Fig. 1), the new species sequences clustered in two different clades. The folicolous species clustered in a same clade (highlighted red in the Fig. 1). The sequence of *Xylariaorientalis* sp. nov. (GMB4701, GMB4705) formed a clade in a sister relation with *X.crinalis* Hai X. Ma, Lar. N. Vassiljeva & Yu Li (FCATAS MHX 751) and *X.filiformis* Hashemi (Alb. & Schwein.) Fr. (GUM IRN 1052) with a low support value. However, this positioning remained consistent across repeated phylogenetic analyses. *Xylariananningensis* sp. nov. (GMB4702, GMB4706) formed a clad in a sister relationship with *X.ficicola* Hai X. Ma, Lar. N. Vassiljeva & Yu Li (HMJAU 22818) with a high support value (BS100/0.96PP). This lineage contains *X.amphithele* San Martin & J. D. Rogers (HAST 529) and *X.jichuanii* sp. nov. (GMB4703, GMB4707). The sequence of *X.taiyangheensis* (GMB4704, GMB4708) formed a separate clade with *X.phyllocharis* Mont. in a sister relationship (BS100/1PP).

### ﻿Taxonomy

#### ﻿ Xylaria jichuanii

Taxon classificationFungiXylarialesXylariaceae

W.Y. Zeng & Q.R. Li sp. nov.

BA154B2C-A975-5BFF-BEEA-42184807F9D3

853632

Fig. 2

##### Etymology.

The epithet “*jichuanii*” pays tribute to the renowned mycologist, Prof. Jichuan Kang, in recognition of his valuable contributions to the field of mycology.

**Figure 2.**
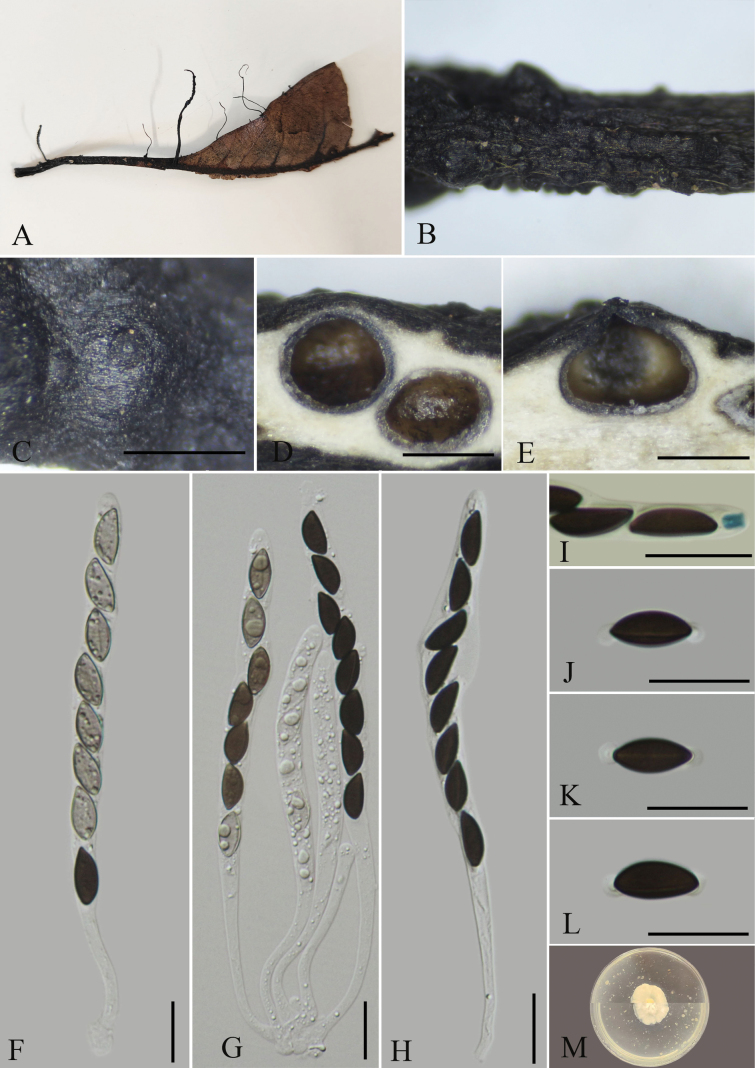
*Xylariajichuanii* (GMB4703) **A** type material **B** stroma **C** surface of stroma **D** transverse section of stroma **E** longitudinal section of stroma **F-H** asci with ascospores **I** a J+, ascus apical apparatus (stained in Melzer’s Reagent) **J–L** ascospores **M** colonies of *Xylariajichuanii* on OA. Scale bars: 0.5 mm (**C–E**); 20 µm (**F–L**).

##### Type.

China. •Guangxi Province, Fangchenggang City, Shiwandashan National Nature Reserve, 21°43'45″N, 107°37'19″E, elev. 459 m, on fallen leaves of unknown plants, August 2023, Wenyu Zeng & Xin Zhou, SWDS1 (GMB4703 Holotype; KUN-HKAS 134915 Isotype; GMBC4703 ex-type).

##### Description.

Saprobic on fallen leaves of an unknown plant. **Sexual morph**: Stromata 1–4.2 cm total length, solitary, upright or prostrate, cylindrical, unbranched, straight to most often sinuous to contorted, the stipe glabrous, 4–18 mm long, the base slightly swollen; fertile part 5–16 × 1–2 mm, cylindrical, surface blackish, with conspicuous to half-exposed perithecial mounds, externally black, interior white. Perithecia 371–776 μm diam., subglobose to globose, texture soft. Ostioles papillate. Asci 84–144 × 5.6–8.6 μm (x̄= 119 × 7.5 μm, n = 30), 8-spored, unitunicate, cylindrical, apically rounded, with a J+, hat-shaped, apical apparatus bluing in Melzer’s reagent, 4–6 × 4–5.5 μm (x̄ = 4.9 × 5.0 μm, n = 30). Ascospores 13.5–17 × 5–8 μm (x̄ = 15.1 × 6.5 μm, n = 30), uniseriate, unicellular, brown to dark brown, ellipsoid to inequilateral, with broadly rounded ends, smooth, with a straight germ slit, equal to the length of the spores, lacking sheath; surrounded with a hyaline sheath swelling at both ends to form papillate non-cellular appendages, sometimes retaining a cellular appendage within a noncellular appendage, epispore smooth. **Asexual morph**: Undetermined.

##### Culture characteristics.

Colonies on OA reaching 1.5–2 mm diam. after 2 weeks at 25 °C, white at first, with irregular margins, then extension spreading toward the edge of the Petri dish; the overall color is light white.

##### Additional material examined.

China. •Guangxi Province, Fangchenggang City, Shiwandashan National Nature Reserve, 21°43'55″N, 107°37'24″E, elev. 482 m, on fallen leaves of unknown plants, August 2023, Wenyu Zeng & Xin Zhou, SWDS1-1 (GMB4707; GMBC4707).

##### Notes.

Phylogenetically, it is closely related to *Xylariaamphithele* and *X.petchii*. However, *X.amphithele* differ from the new collection by its smaller (≤50 mm) stromata which are conical to globose at the fertile part, large asci 130–220 μm in total length with the spore-bearing part being 80–120 μm long. Furthermore, *X.amphithele* has a smaller apical apparatus (3.5–4.5 × 2–3 μm), and smaller perithecia (300–550 μm broad) (Ju and Hsieh 2023). *Xylariapetchii* differs by having a conical to subglobose fertile part (1–10 × 0.5–2 mm), composed of clusters of perithecia near the top of the stromata, smaller perithecia (250–550 µm in diameter), and smaller ascospores (7.5–10 × 3.5–5 µm) with a sigmoid germ slit (Ju and Hsieh 2023). Morphologically, it is closely related to *X.ficicola*, which can be easily distinguished from *X.jichuanii* by its conical to subglobose capitate fertile head, and larger apical apparatus measuring 5–7.5 × 3–3.5 µm. Additionally, it possesses larger ascospores (16–22.7 × 6.5–8.5 µm) with a round hyaline noncellular appendage of up to 5 × 5 μm (Ma et al. 2011).

#### ﻿ Xylaria nanningensis

Taxon classificationFungiXylarialesXylariaceae

W.Y. Zeng & Q.R. Li sp. nov.

3366CEC3-15BC-5791-ACD6-54C242749787

853633

Fig. 3

##### Etymology.

The specific epithet refers to its collection location, Nanning City.

##### Type.

China. •Guangxi Province, Nanning City, Liangfengjiang National For-est Park, 22°43'28″N, 108°16'59″E, elev. 97 m, on fallen leaves of unknown plants, August 2023, Wenyu Zeng & Xin Zhou, LFJ1 (GMB4702 Holotype; KUN -HKAS 134916 Isotype; GMBC4702 ex-type).

**Figure 3.**
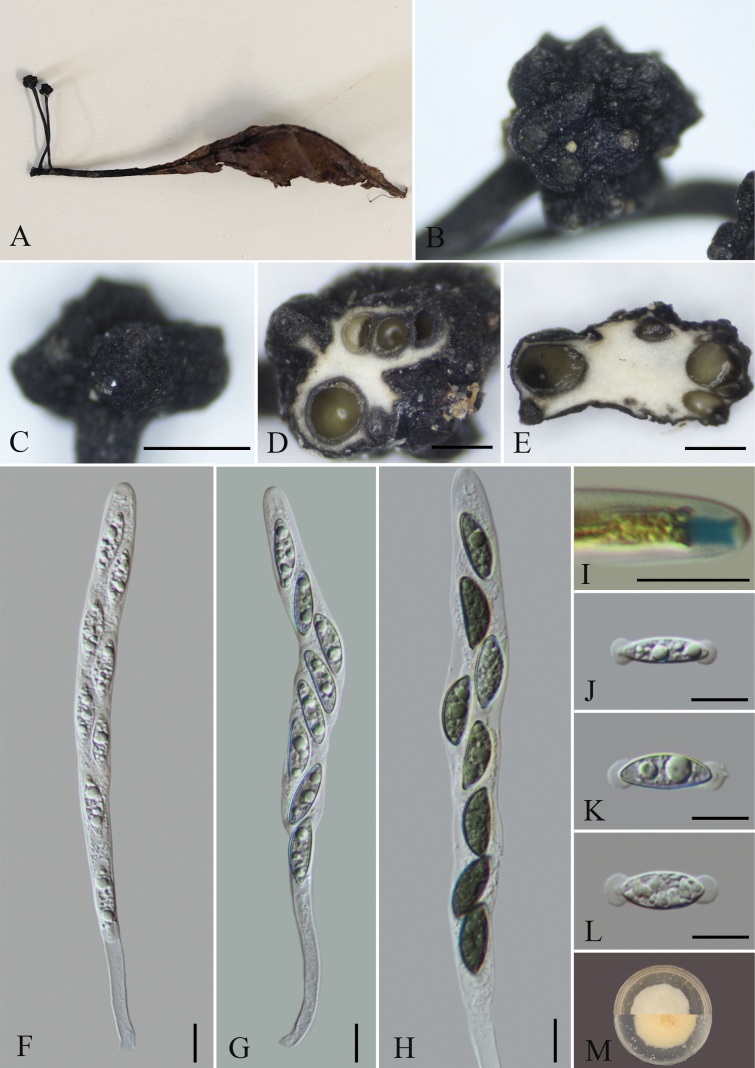
*Xylariananningensis* (GMB4702) **A** type material **B** fertile part of stroma **C** surface of stroma **D** transverse section of stroma **E** longitudinal section of stroma **F–H** asci with ascospores **I** a J+, ascus apical apparatus (stained in Melzer’s Reagent) **J–L** ascospores **M** colonies of *Xylariananningensis* on OA. Scale bars: 0.5 mm (**C–E**); 10 µm (**F–L**).

##### Description.

Saprobic on fallen leaves of unknown plants. **Sexual morph**: Stromata 1.1–2.8 cm in total length, solitary, upright, unbranched, with capitate fertile apex, head conical to subglobose 0.5–1.2 mm diam × 0.5–1.8 mm thick, consists of closely packed perithecia, surface black, rounded, on a long stipe, slightly swollen at base, rough. Externally black, interior white, texture soft. Stipes thin, glabrous, up to 2 cm long. Perithecia 85–395 μm diam., oval to spherical, embedded, closely arranged, interior white. Ostioles slightly papillate. Asci 114–156 × 6.8–13.6 μm (x̄ = 135 × 10.2 μm, n = 30), 8-spored, unitunicate, cylindrical, apically rounded, with an inverted hat shaped apical apparatus, blue staining in Melzer’s reagent, 5.2–5.7 × 4.9–5.4 μm (x̄ = 5.5 × 5.2 μm, n = 30). Ascospores 15.0–24.1 × 4.4–7.4 μm (x̄ = 19.0 × 5.5 μm, n = 30), uniseriate, unicellular, hyaline when immature, slight brown to dark brown at maturity, ellipsoid-inequilateral, with rounded ends, smooth, each end surround a round sheath, up to 5 × 5 μm, lacking germ slit; epispore smooth. **Asexual morph**: Undetermined.

##### Culture characteristics.

Colonies on OA reaching 3–4 cm diam. after 2 weeks at 25 °C, white at first, with irregular margins, then extension spreading toward the edge of the Petri dish; the overall color is light white.

##### Additional material examined.

China. •Guangxi Province, Nanning City, Liangfengjiang National Forest Park, 22°43'28″N, 108°16'59″E, elev. 207 m, on fallen leaves of unknown plants, August 2023, Wenyu Zeng & Xin Zhou, LFJ1-1 (GMB4706; GMBC4706).

##### Notes.

In the phylogram (Fig. 1), it is closely related to *X.ficicola*. The latter can be distinguished by the size of stromata stipe, asci and ascospores (Pan et al. 2022). *Xylariananningensis* has less than 2 cm long stipe (vs. up to 6 cm long in *X.ficicola*), asci measuring 114–156 × 6.8–13.6 μm (vs. 190–220 × 8–10 μm in *X.ficicola*) and ascospores measuring 15.0–24.1 × 4.4–7.4 μm (vs. 16–22.7 × 6.5–8.5 μm in *X.ficicola*). Moreover, the apical apparatus of the new species is slightly smaller (5.2–5.7 × 4.9–5.4 μm vs. 5–7.5 × 3–3.5 μm) (Ma et al. 2011). Morphologically, *X.nanningensis* is similar to *X.guazumae* F. San Martín & J.D. Rogers (San and Rogers 1989), but the latter grows on the fallen fruits of *Guazumaulmifolia* Lam. (Sterculiaceae) and has relatively smaller ascospores (14–19 × 5.5–6 μm) and smaller rectangular apical ring (2.8–3.2 × 2–2.5 μm) (San and Rogers 1989). Another morphologically close species is *X.polysporicola* Hai X. Ma & X.Y. Pan, but the latter can be distinguished by its stromata which has acute sterile apex up to 2 mm long, fertile part 2–15 mm long × 0.5–1.6 mm diam. (vs. fertile apex 0.5–1.2 mm long × 0.5–1 mm diam), and smaller ascospores (11.5–15 × 5.5–8 µm) with a straight germ slit (Pan et al. 2022).

#### ﻿ Xylaria orientalis

Taxon classificationFungiXylarialesXylariaceae

W.Y. Zeng & Q.R. Li sp. nov.

C6EB6969-B82E-5A9E-8075-76F546F4ADF3

853634

Fig. 4

##### Etymology.

The specific epithet refers to the name of host, *Hedyosmumorientalis* Merr. & Chun.

##### Type.

China. •Guangxi Province, Rongshui County, Jiuwanshan National Na-ture Reserve, 25°10'42″N, 108°44'58″E, elev. 984 m, on fallen leaves of *Hedyosmumorientalis* (Chloranthaceae), August 2023, Wenyu Zeng & Xin Zhou, JWS2 (GMB4701 Holotype; KUN-HKAS 134917 Isotype; GMBC4701 ex-type).

**Figure 4.**
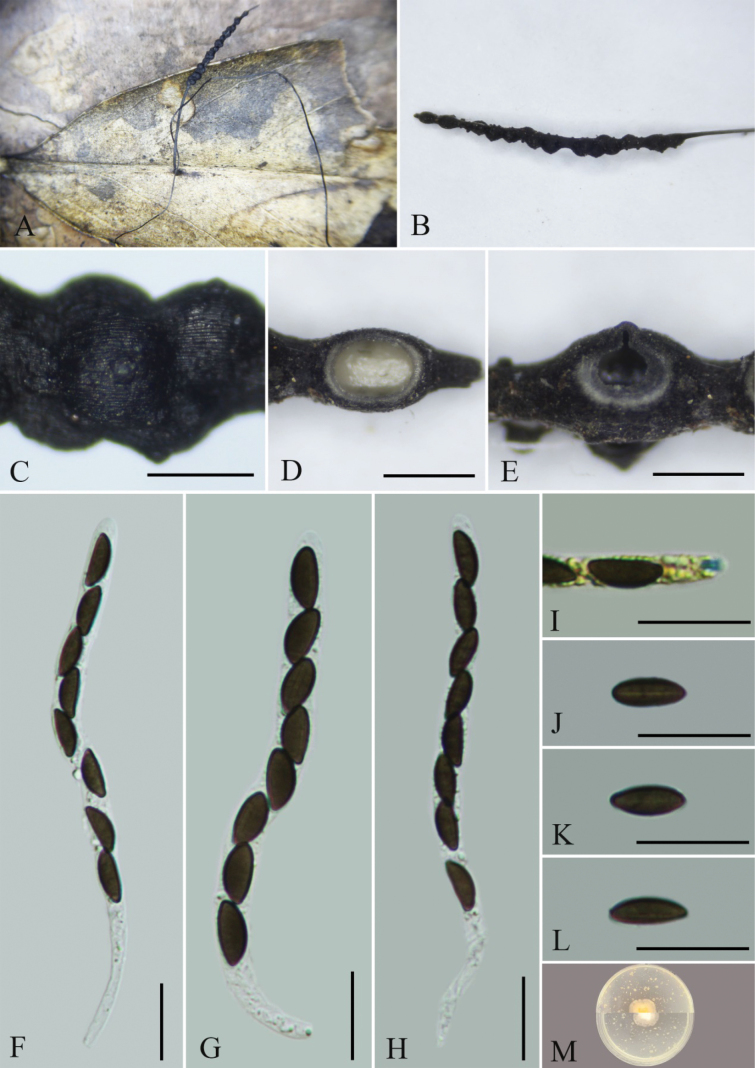
*Xylariaorientalis* (GMB4701) **A** type material **B** stroma **C** surface of stroma **D** transverse section of stroma **E** longitudinal section of stroma **F-H** asci with ascospores **I** a J+, ascus apical apparatus (stained in Melzer’s Reagent) **J–L** ascospores **M** colonies of *Xylariaorientalis* on OA. Scale bars: 0.5 mm (**C–E**); 15 µm (**F–L**).

##### Description.

Saprobic on fallen leaves of *Hedyosmumorientalis*. **Sexual morph**: Stromata 1.7–8.5 cm long, upright, solitary, black, thread-like, unbranched, with a long sterile filiform apex up to 0.2–4 cm long, fertile part 4–13 mm long × 0.5–1 mm wide, glabrous, finely longitudinally striate, the base slightly swollen, with 2/3-exposed to fully exposed perithecial mounds. The exterior is black, interior white, has soft texture. Perithecia subglobose, 350–573 μm diam. Ostioles papillate. Asci 81–115 × 4.6–6.6 μm (x̄ = 98 × 5.6 μm, n = 30), 8-spored, unitunicate, cylindrical, with a J+, tubular to wedge-shaped apical apparatus bluing in Melzer’s reagent, 2.1–4.1 × 2.8–4.0 μm (x̄ = 3.1 × 3.4 μm, n = 30). Ascospores 9.4–11.2 × 3.5–5.0 μm (x̄ = 10.2 × 4.3 μm, n = 30), uniseriate, unicellular, dark brown to black, ellipsoid-inequilateral, with narrowly rounded ends, smooth, straight germ slit of a spore-length, lacking sheath, epispore smooth. **Asexual morph**: Undetermined.

##### Culture characteristics.

Colonies on OA reaching 12–15 mm diam. after 2 weeks at 25 °C, white at first, with irregular margins, then extension spreading toward the edge of the Petri dish; the overall color is light white.

##### Additional material examined.

China. •Guangxi Province, Rongshui County, Jiuwanshan National Nature Reserve, 25°10'42″N, 108°44'58″E, elev. 765 m, on fallen leaves of *Hedyosmumorientalis*, August 2023, Wenyu Zeng & Xin Zhou, JWS2-1 (GMB4705; GMBC4705).

##### Notes.

Morphologically and phylogenetically (Fig. 1), *Xylariaorientalis* is closely related to *X.filiformis* and *X.crinalis*, sharing similar stromatal characteristics (Hashemi et al. 2014; Ma and Yu 2018). However, differences are observed in their asci and ascospores. The ascospores of *X.orientalis* are distinctly dark brown to black, whereas those of *X.filiformis* are light brown. Additionally, *X.filiformis* has larger asci 130–155 × 5.5–6.2 μm, and ascospores 12.5–21 × 4.5–5.5 μm (Hashemi et al. 2014). *Xylariacrinalis* differs by having a few scattered perithecia on the stroma, a more or less conspicuous germ slit, a narrower apical apparatus 1.5–2.5 µm broad, larger ascospores 14–17.5 × 3.5–6 µm, and its occurrence on wood. Morphologically, *Xylariaorientalis* also shares stromatal characteristics with *X.vagans* Petch. However, *X.vagans* is distinguished by its black rhizomorphoid mycelium connecting dead leaves, asci lacking a J+ apical apparatus, and ascospores with broadly rounded ends that lack a germ slit (Ju and Hsieh 2023).

#### ﻿ Xylaria taiyangheensis

Taxon classificationFungiXylarialesXylariaceae

W.Y. Zeng & Q.R. Li sp. nov.

0E3B27A6-69A0-57B7-B132-E69A718F386A

853635

Fig. 5

##### Etymology.

The specific epithet refers to its collection location, Taiyanghe Provincial Nature Reserve.

##### Type.

China. •Yunnan Province, Puer City, Taiyanghe Provincial Nature Reserve, 22°38'24″N, 101°15'13″E, elev. 1032 m, on twigs of *Goniothalamuscheliensis* Hu, July 2023, Wenyu Zeng & Xin Zhou, TYH5 (GMB4704 Holotype; KUN-HKAS 134918 Isotype; GMBC4704 ex-type).

**Figure 5.**
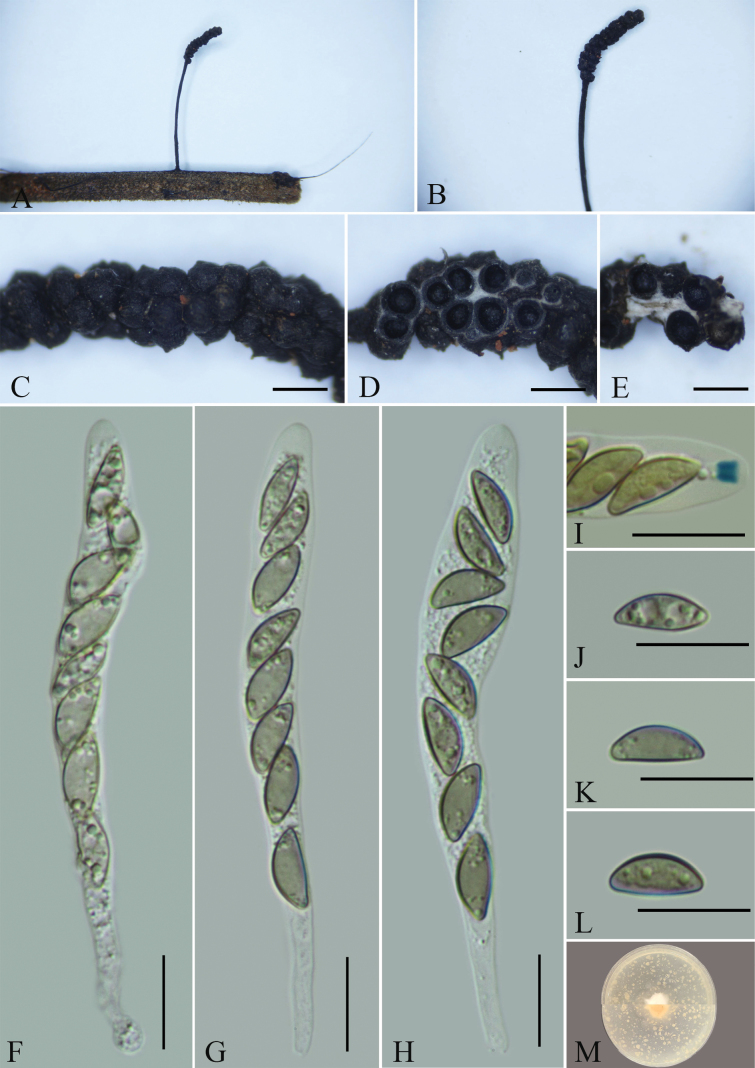
*Xylariataiyangheensis* (GMB4704) **A** type material **B** stroma **C** surface of stroma **D** transverse section of stroma **E** longitudinal section of stroma **F–H** asci with ascospores **I** a J+, ascus apical apparatus (stained in Melzer’s Reagent) **J–L** ascospores **M** colonies of *Xylariataiyangheensis* on OA. Scale bars: 0.5 mm (**C–E**); 15 µm (**F–L**).

##### Description.

Saprobic on twigs of *Goniothalamuscheliensis*. **Sexual morph**: Stromata 0.7–2.7 cm in total length, cylindrical at fertile part, unbranched, long and smooth stipe, 10–24 mm long, fertile part 5–13 mm long × 0.5–1 mm diam., surface black, with 2/3-exposed to fully exposed perithecial mounds, interior white, homogeneous. Perithecia 247–365 μm broad, closely arranged. Ostioles papillate. Asci 79–107 × 6.5–11.3 μm (x̄ = 93 × 8.9 μm, n = 30), 8-spored, unitunicate, cylindrical, apically rounded, with a urn-shaped apical apparatus, blue staining in Melzer’s reagent, 4.1–7.1 × 4.6–6.5 μm (x̄ = 5.6 × 5.5 μm, n = 30). Ascospores 11.6–20.3 × 4.0–7.0 μm (x̄ = 15.9 × 5.5 μm, n = 30), uniseriate, unicellular, brown, ellipsoid to inequilateral, with broadly rounded ends, smooth, without germ slit, lacking appendages and sheaths, epispore smooth. **Asexual morph**: Undetermined.

##### Culture characteristics.

Colonies on OA reaching 1–1.5 cm diam. after 2 weeks at 25 °C white at first, with irregular margins, then extension spreading toward the edge of the petri dish; the overall color is light white.

##### Additional material examined.

China. •Yunnan Province, Puer City, Taiyanghe Provincial Nature Reserve, 22°38'24″N, 101°15'13″E, elev. 962 m, on twigs of unknown plants, July 2023, Wenyu Zeng & Xin Zhou, TYH5-1 (GMB4708; GMBC4708).

##### Notes.

Phylogenetically, it is closely related to *X.phyllocharis*. However, *X.phyllocharis* differs in having stromata with a slight perithecial mound, smaller ascomata (100–250 μm in breadth), and smaller ascospores (9–12.5 × 4.5–7 μm) with a straight germ slit (Ju and Hsieh 2023). Morphologically, *X.taiyangheensis* and *X.noduliformis* show similar stromata, but the latter has smaller ascospores (9–11 × 5.5–7 µm) with a straight germ slit (Ju and Hsieh 2023). Moreover, *X.noduliformis* differs from the new collection by its host, and association with petioles.

## ﻿Discussion

The phylogenetic tree (Fig. 1) reveals that *Xylaria* does not share a common ancestor and evolved from different ancestral lineages. The *Xylaria* species positions in the phylogram (Fig. 1) are consistent with previous studies (Hsieh et al. 2010; U’Ren et al. 2016; Ma et al. 2022a; Li et al. 2024a).

Phylogenetic analysis in this study reveals that *Xylariajichuanii*, *X.nanningensis*, *X.orientalis* and *X.taiyangheensis* form a distinct cluster within the phylogenetic tree. The three folicolous species of this study *Xylariajichuanii*, *X.nanningensis*, and *X.orientalis* clustered with *X.amphithele*, *X.crinalis*, *X.filiformis*, *X.ficicola*, *X.diaoluoshanensis*, *X.fulvotomentosa*, *X.hedyosmicola*, *X.petchii*, *X.polysporicola* and *Entalbostromaerumpens*, all of which are related to fallen leaves, except for *X.crinalis* which is wood-inhabiting (Pan et al. 2024). The wood-inhabiting species of this study *X.taiyangheensis* clustered with the leaf-inhabiting *X.phyllocharis*, forming a distinct clade in the phylogram, highlighting the possible influence of substrate on the evolution of these taxa. These findings corroborate the conclusions of Ju and Hsieh (2023) and Pan et al. (2024), affirming consistency in systematic analysis results. This may indicate that species of *Xylaria*, which associated on plant leaves and petioles, may have a similar evolutionary process.

The fallen leaves and petioles serve as a growth substrate for some *Xylaria* species. However, these species are often overlooked because of their small stromata. They typically have a small number of stromata, and multiple species may grow on the same leaf, making collection challenging (Ju and Hsieh 2023; Pan et al. 2024). *Xylariapetchii* C. G. Lloyd, *X.diaoluoshanensis* Xiao Y. Pan and *X.fulvotomentosa* Xiao Y. Pan, on the fallen leaves were introduced within Hainan tropical rainforest national park (Pan et al. 2024). Ju and Hsieh (2023) provided a compilation of *Xylaria* species associated with fallen leaves and petioles worldwide, as well as new species. A total of 44 *Xylaria* species found on fallen leaves and petioles have been officially documented globally. To date, fourteen taxa, including *X.betulicola* Hai X. Ma, Lar.N. Vassiljeva & Yu Li, *X.diminuta* F. San Martín & J.D. Rogers, *X.ficicola* Hai X. Ma, Lar.N. Vassiljeva & Yu Li, *X.foliicola* G. Huang & L. Guo, *X.aristata* Mont. *var. aristata*, *X.hedyosmicola* Hai X.Ma & X.Y. Pan, *X.simplicissima* (Pers.) Y.M. Ju & H.M. Hsieh, *X.lindericola* Hai X. Ma & X.Y. Pan, *X.polysporicola* Hai X. Ma & X.Y. Pan, X.siculaPass. & Beltr.f.major Ciccarone, *X.minuscula* Y.M. Ju & H.M. Hsieh, *X.diaoluoshanensis*, *X.fulvotomentosa* and *X.petchii* have been discovered on fallen leaves in China (Hsieh et al. 2010; Ma et al. 2011; Zhu and Guo 2011; Huang et al. 2014, 2015; Ma et al. 2022a; Ju and Hsieh 2023; Pan et al. 2024). Here three new *Xylaria* species associated on fallen leaves and petioles were introduced. Recent publications have shown that a high number of *Xylaria* species associated with fallen leaves in tropical and subtropical regions, with most findings confirming new species (Ju and Hsieh 2023; Pan et al. 2024). A more thorough examination of *Xylaria* species growing on leaves and petioles is needed.

The southwestern region of China boasts the world’s largest karst habitat area, harbouring abundant and distinctive fungal species. Recent reports of new *Xylaria* species from China, particularly from Karst regions, underscore the significance of these environments as focal points of fungal diversity (Li et al. 2024a; Zhu et al. 2024). Pan et al. (2022, 2024), Li et al. (2024a, 2024b) and Zhu et al. (2024) reported more than 80 new species of Xylariales include 45 *Xylaria* species from the karst region of China. Four new pale-spored species of *Xylaria* were introduced from Southwest China (Ma et al. 2022a). Zhu et al. (2024) reported two new species, *X.aleuriticola* and *X.microcarpa* on fallen fruits, and one new record from south China. Through examination of fallen leaves and twigs of plants in these unique habitats, we identified four new *Xylaria* species from the Karst regions of Yunnan and Guangxi provinces, China. This extensive documentation underscores the remarkable diversity and ecological significance of the Xylariales in karst environments. These findings not only broaden our comprehension of *Xylaria* diversity in the region but also underscores the importance of continuous exploration and documentation efforts, particularly in ecologically distinctive areas such as the Karst Region in South China.

## Supplementary Material

XML Treatment for Xylaria jichuanii

XML Treatment for Xylaria nanningensis

XML Treatment for Xylaria orientalis

XML Treatment for Xylaria taiyangheensis

## ﻿Additional information

### Author ORCIDs

Wenyu Zeng https://orcid.org/0009-0001-6826-7591

Kamran Habib https://orcid.org/0000-0003-2572-0306

Xin Zhou https://orcid.org/0009-0002-4204-1225

Yulin Ren https://orcid.org/0009-0003-9063-425X

Xiangchun Shen https://orcid.org/0000-0002-4333-9106

Yingqian Kang https://orcid.org/0000-0003-0189-9655

Jichuan Kang https://orcid.org/0000-0002-6294-5793

Qirui Li https://orcid.org/0000-0001-8735-2890

### Data availability

The datasets generated during and/or analyzed during the current study are available in the MycoBank repository (included in the manuscript), and GenBank (included in Table 1). And also, the datasets generated during and/or analyzed during the current study are available from the corresponding author on reasonable request.

## Supplementary materials

Supplementary material 1 Alignment file Data type fasta https://binary.pensoft.net/file/1127995This dataset is made available under the Open Database License (http://opendatacommons.org/licenses/odbl/1.0/). The Open Database License (ODbL) is a license agreement intended to allow users to freely share, modify, and use this Dataset while maintaining this same freedom for others, provided that the original source and author(s) are credited.Wenyu Zeng, Kamran Habib, Xin Zhou, Yulin Ren, Xiangchun Shen, Bei Wang, Yingqian Kang, Jichuan Kang, Qirui Li
